# Nanoscale Topical Pharmacotherapy in Management of Psoriasis: Contemporary Research and Scope

**DOI:** 10.3390/jfb14010019

**Published:** 2022-12-29

**Authors:** Mohammad Zaki Ahmad, Abdul Aleem Mohammed, Mohammed S. Algahtani, Awanish Mishra, Javed Ahmad

**Affiliations:** 1 Department of Pharmaceutics, College of Pharmacy, Najran University, Najran 11001, Saudi Arabia; 2 Department of Pharmacology and Toxicology, National Institute of Pharmaceutical Education and Research (NIPER), Guwahati 781101, Assam, India

**Keywords:** psoriasis, pathophysiology, nanomedicine, topical delivery, targeted pharmacotherapy

## Abstract

Psoriasis is a typical dermal condition that has been anticipated since prehistoric times when it was mistakenly implicit in being a variant of leprosy. It is an atypical organ-specific autoimmune disorder, which is triggered by the activation of T-cells and/or B-cells. Until now, the pathophysiology of this disease is not completely explicated and still, many research investigations are ongoing. Different approaches have been investigated to treat this dreadful skin disease using various anti-psoriatic drugs of different modes of action through smart drug-delivery systems. Nevertheless, there is no ideal therapy for a complete cure of psoriasis owing to the dearth of an ideal drug-delivery system for anti-psoriatic drugs. The conventional pharmacotherapy approaches for the treatment of psoriasis demand various classes of anti-psoriatic drugs with optimum benefit/risk ratio and insignificant untoward effects. The advancement in nanoscale drug delivery had a great impact on the establishment of a nanomedicine-based therapy for better management of psoriasis in recent times. Nanodrug carriers are exploited to design and develop nanomedicine-based therapy for psoriasis. It has a promising future in the improvement of the therapeutic efficacy of conventional anti-psoriatic drugs. The present manuscript aims to discuss the pathophysiology, conventional pharmacotherapy, and contemporary research in the area of nanoscale topical drug delivery systems for better management of psoriasis including the significance of targeted pharmacotherapy in psoriasis.

## 1. Introduction

**Figure 1:**
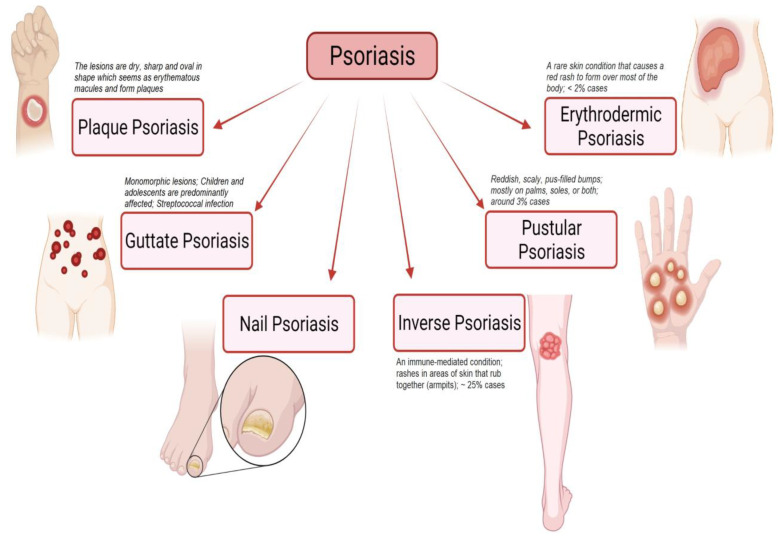
Schematic illustration highlights different types of psoriasis (such as plaque psoriasis, guttate psoriasis, nail psoriasis, inverse psoriasis, pustular psoriasis, and erythrodermic psoriasis) and their characteristic features. “Image created with BioRender.com”.

Psoriasis is a non-contagious autoimmune skin disorder characterized by inflammatory conditions of reverting occurrence with erythematous scaly skin lesions and hyperkeratosis plaques [1,2,3]. Psoriasis appears on the skin as patches due to the pile-up of skin cells on the surface of the skin. The International Federation of Psoriasis Association reported that psoriasis affects 2 to 3% of the world’s population [4]. The chronic effect of psoriasis can develop psoriatic arthritis in 10–30% of the cases [4]. Psoriatic arthritis is inflammatory arthritis leading to erosions of articulate cartilage and consequently sustained inflammation results in irreversible joint destruction. The American Academy of Dermatology has classified psoriasis based on the extent of an inflammatory process, patient’s severity, and rash localization as plaque psoriasis (psoriasis vulgaris), eruptive psoriasis, pustular, and erythrodermic (exfoliative psoriasis), and inverse psoriasis or intertriginous psoriasis (illustrated in Figure 1) [2,5,6]. Around 80% of the psoriatic populations are affected by the plaque psoriasis type exhibiting itchy and painful red skin lesions with silvery scales that can be noticed on all body parts including the oral cavity and genital organs [7,8,9]. Based on the degree of psoriasis coverage extent on the body, The National Psoriasis Foundation defines psoriasis as mild, moderate, and severe where psoriasis affecting less than 3% of the body, 3% to 10% and more than 10%, respectively [7]. Moreover, the influence of psoriatic lesions on a patient’s quality of life is also used as a basis to quantify the severity of psoriasis. The abstruse impact has been experienced by moderate to severe psoriasis patients on the quality of their everyday life.

The etiology of psoriasis is unclear. The under-recognized cause may be a combination of certain types of microbial infection, genetics, and or environmental factors [1,2,3,7,9]. Various non-specific triggers, like mild trauma (tattoos, piercings, and scratching), chemical irritants, and/or sunburn, may provoke psoriasis [5]. Similarly, psoriasis can be aggravated by the use of drugs such as β-blockers, lithium, antimalarial, and nonsteroidal anti-inflammatory drugs (NSAIDs) [5,10]. In the immune system, the function of T-cells is to hunt down the invading foreign organisms and provide the antibody response to keep the body healthy [1,2,3,7,9]. In psoriasis, the T-cells recognize some patches of the body’s skin as a foreign body and attack it. This attack leads to the overproduction of new T-cells, skin cells, and white blood cells [7,9]. The accumulation of dead skin cells creates hallmark scaly patches as seen in psoriatic lesions. According to the Mayo Clinic, there is a greater risk of psoriasis appearance in HIV patients, and children with frequently infected throats or other recurring infections [7]. As psoriasis often begins in the skin folds of the body, obese persons are at a higher risk. In addition, an obese person with smoking habits is also highly susceptible to psoriasis. Flare-ups may be precipitated by stress and triggered by certain drugs. These include beta-blockers, interferon, and lithium [7]. The physical and psychological stigma of psoriasis has a moderate to high impact on the life quality of psoriatic patients [11]. Approximately 60% of psoriatic patients and about 40% of psoriatic arthritis patients stated that the diseases affect their daily life psychologically and physically as well [4]. The psoriasis area and the severity index (PASI) scores are parameters for the diagnosis and can be employed to quantify the severity of the disease [5,12]. Psoriasis screening tools such as the Toronto psoriatic arthritic screening (ToPAS) questionnaire, psoriasis epidemiology screening tool (PEST), and psoriatic arthritis screening and evaluation (PASE) questionnaire can be used to aid diagnosis [5,13]. Psoriasis shows characteristic changes in skin histopathology such as epidermal acanthosis, hyperkeratosis, and parakeratosis [5]. In the dermis, the tips of dermal papillae exhibited contorted and dilated blood vessels. The most variant diagnosis of psoriasis includes tinea capitis and tinea corporis, seborrheic dermatitis, and eczema [5]. 

In recent years, the advancement in the nanoscale drug delivery approach had a great impact on the establishment of a nanomedicine-based therapy for better management of psoriasis. Nano drug-carrier systems are exploited to design and develop nanoscale drug delivery (nanomedicine-based therapy) to improve the therapeutic efficacy of loaded drugs in psoriasis. The present review aims to discuss the pathophysiology, current pharmacotherapy in clinical practice, and contemporary research in the area of nanoscale drug delivery systems for the better management of psoriasis including the significance of targeted pharmacotherapy in psoriasis.

## 2. Pathophysiology and Molecular Basis of Psoriasis

The pathophysiology of psoriasis is poorly understood [14]. Through immune system contribution, the involvement of the immune system in the eruption of psoriasis is now widely accepted [5,15,16]. The pathological advancement of psoriasis is based on a sequence of coherent events that involves the stimulation of circulating immune cells and their signaling molecules like chemokines, cytokines, and growth factors [2]. All these factors further lead to marked hyperkeratosis, congealing of the epidermis, and neovascularization. Epidermal acanthosis is the proliferation and the abnormal accelerated differentiation of keratinocytes observed in the histological examination of psoriatic skin [2,5]. In the psoriatic epidermis, keratinocytes proliferate and mature rapidly, and the terminal differentiation of squamous corneocytes is incomplete [17]. The stratum corneum (SC) of the psoriatic lesions shows hyperkeratosis and parakeratosis which are characterized by skin thickening and abnormal maturation [1,18]. Due to the abnormal maturation, SC aberrantly retains intact nuclei and releases a few extracellular lipids that typically cement the adhesion of corneocytes [17,18]. Consequently, poorly adherent SC is formed and this results in typical flakes or scales of psoriasis lesions [17,18]. Further, keratinocytes also produce vascular endothelial growth factors (VEGF), and platelet-derived growth factors (PDGF) leading to the formation and growth of blood vessels [1,19,20]. The activation of endothelial cells leads to the production of keratinocytes growth factor (KGF) which promotes keratinocytes proliferation and vasodilation of dermis capillaries, thereby granting the extravasation of circulating immune cells [21].

**Figure 2:**
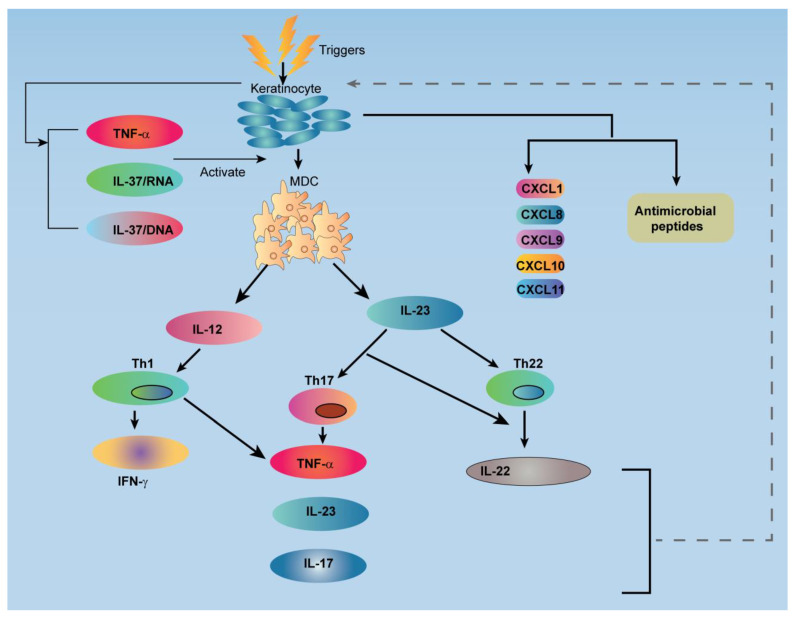
Schematic illustration shows a relationship between the T-cells and the dendritic cells (DC) involved in the underlying pathology of psoriasis. TNF: Tumor Necrosis Factor; IL: Interleukin; MDC: Myeloid Dendritic Cell; Th: T-helper; IFN: Interferon; CXCL: Chemokine (C-X-C motif) Ligand.

The interaction between keratinocytes and mononuclear leucocytes is responsible for the eruption of the psoriatic lesion [21]. Psoriasis susceptibility genes influence the gene expression program in these diverse cell types [21,22]. Keratinocytes actively participate in the recruitment and activation of leukocytes in the psoriatic lesion [21,22]. A balanced cross-talk between the activation of innate and adaptive immune cell, and different factor produced by keratinocytes directly affect the T-cells and dendritic cells (DCs) [1,2,21,22]. The relationship between the T-cells and the DCs involved in the underlying pathology of psoriasis is shown in Figure 2.

The high level of tumor necrosis factor (TNF) and inducible nitric oxide synthase (iNOS) is expressed by CD11c^+^ DCs in psoriatic lesions [18,21,23,24,25]. Furthermore, CD11c^+^ DCs also produces cytokines IL-20 and IL-23, which in turn, potentially activate the keratinocytes and T cells, respectively [18,23,24,25]. The activation of keratinocytes by tissue necrosis factor α (TNFα) and interferon γ (INFγ) leads to the secretion of different types of cytokines (MCP-1, IL-8, CXCL1, CXCL2, CXCL3, CCL20, CXCL9, CXCL10, CXCL11), these cytokines attract many leukocytes (monocytes, neutrophils, PDCs, CCR6+, Th1, CXCR3+, Th1) from the systemic circulation to the skin area [1,17,21,22]. Further, these cytokines participate in amplifying and maintaining the inflammation response that provokes lesions. Interleukin 17 (IL-17), interleukin 22 (IL-22), and INFγ are involved in abnormal proliferation and epidermal hyperplasia [1,21,22,26]. Another important factor responsible for triggering the psoriatic lesion is the production of interferon-α (INF-α) from BDCS-2^+^ CD123^+^plasmacytoid DCs [21,27]. DC-LAMP or CD83 represent fractional maturation markers for CD11c^+^DCs and function as conventional DCs in terms of presenting antigens to T cells for the triggering of the acquired immune response [21,23]. Cytotoxic T cells (CD8+ T) have a great source of pro-inflammatory cytokines (INF-γ), IL-13, IL-22, and IL-17) [1,28]. IL-13 induces the expression of CCL22 in keratinocytes and matrix metalloproteinase-9 activation, leading to the migration of leukocytes into the epidermis [1,29,30].

## 3. Current Pharmacotherapy for the Management of Psoriasis

**Figure 3:**
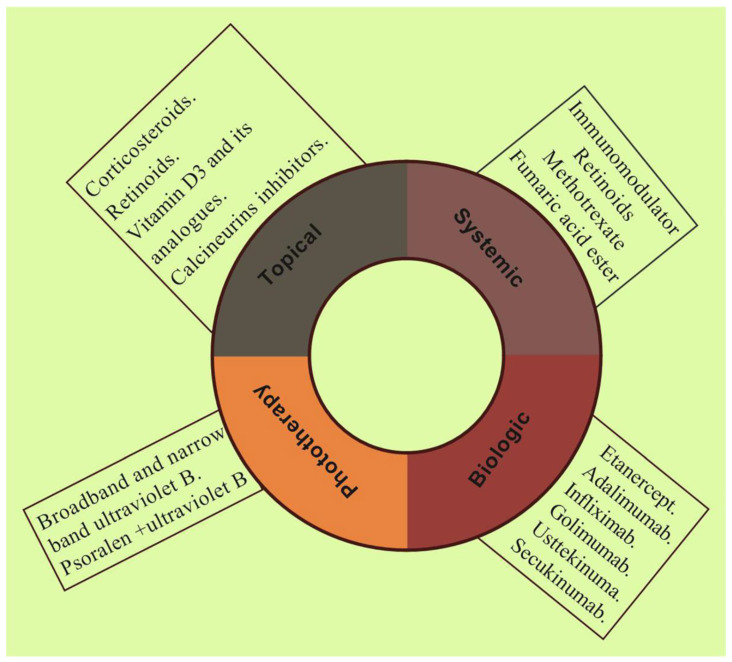
Schematic illustration shows different pharmacotherapy such as topical, systemic, biologic, and phototherapy employing various therapeutics in current clinical practice for the management of psoriasis.

At present, there is no ideal pharmacotherapy for the complete cure of psoriasis. The present treatment option primarily aims to subside the symptoms and avert the disease progression and improve the patient’s quality of life. The available treatment options in the management of psoriasis include calcineurin inhibitors, vitamin D3 analogues, keratolytic agents, corticosteroids, retinoids, and biologics. The available pharmacotherapy and its applications as topical, systemic, and phototherapy in the management of psoriasis are illustrated in Figure 3. 

Further details on available drug therapy with associated characteristics and their drug action mechanism utilized in the management of psoriasis are summarized in Table 1.

### Combination Approach for Management of Psoriasis

Combination therapy, also called polytherapy, is the use of multiple medications or therapies in order to combat the same condition, providing low-dose, toxicity-sparing regimens of each therapeutic agent [51,52]. Different psoriasis treatments have different modes of action. Together, they can fight psoriasis on multiple fronts, and the effect is improved [7]. This approach can decrease the associated side effects and relieve the symptoms more quickly and efficiently. The combination therapies which are widely employed are methotrexate with ultraviolet B (UVB), ultraviolet A(PUVA) and UVB, acitretin with ultraviolet B (UVB) and psoralen plus ultraviolet A (PUVA), and methotrexate with cyclosporine [52]. Table 2 list the reported combination therapy for psoriasis. Another strategy for combination treatment is rotational therapy which is a practical approach to decrease the cumulative toxicity of anti-psoriasis treatments [53]. In this effort, patients are moved from one therapy to another therapy to minimize overall dosage and anticipated toxicities [54,55].

## 4. Challenges in Topical Delivery of Anti-Psoriasis Drugs

Drug delivery through the skin is one of the significant obstacles to obtaining the optimum therapeutic efficacy of the applied drugs. The stratum corneum (SC) is the outermost layer of the skin which acts as an active wall inhibiting the penetration of xenobiotics [82,83]. The most important factor associated with the percutaneous absorption of drugs is the degree of hydration of SC [82,83]. Rigidization of the normal skin, which is the absence of a normal moisturizing factor, is a common problem associated with psoriatic skin [83]. Various approaches have been tested to deliver different drugs through the skin. Nevertheless, topical administration of semisolid formulation faces considerable impediments because of low skin penetration, rapid degradation of actives or volatilization of a volatile compound, and photodegradation. 

## 5. Nanoscale Topical Pharmacotherapy in Effective Treatment of Psoriasis

**Figure 4:**
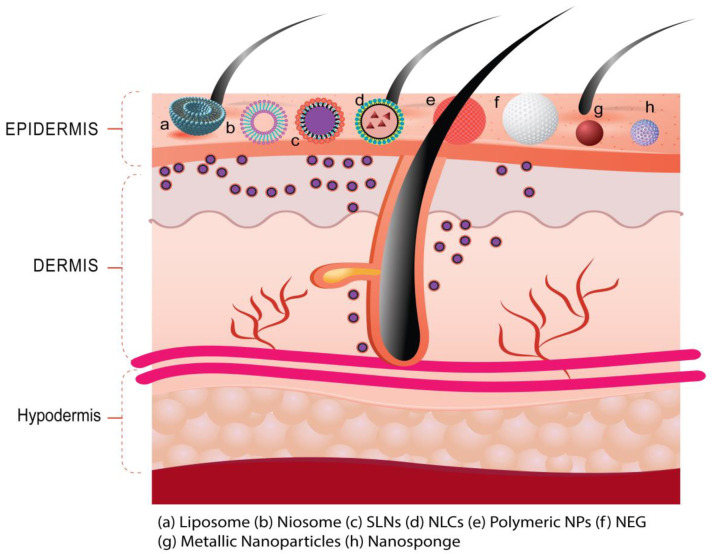
Nanoscale delivery of loaded therapeutics utilizing different types of drug carrier systems (such as liposome, niosome, SLNs, NLCs, polymeric NPs, metallic NPs, etc.) for deeper skin penetration and local targeting helpful to improve the therapeutic efficacy in psoriasis.

The nanotechnology-mediated pharmacotherapy provides propitious skin interactions as expected in psoriasis. The topical administration of nanoscale delivery systems combines the advantages of deeper skin penetration, due to increased surface area, as well as the local targeting that is illustrated in Figure 4. It involved the delivery of loaded therapeutics employing nanodrug carrier systems of polymeric, lipidic, and inorganic in nature [1]. The nanodrug carrier system prepared from the polymer is a nanoparticulate system of natural/synthetic origin and biodegradable/non-biodegradable in nature. In addition, nanodrug carrier systems prepared from lipids are nanoparticulate/nanovesicular systems of natural/synthetic lipids of biodegradable/non-biodegradable nature [2]. Furthermore, nanodrug carrier systems prepared from inorganic materials are nanoparticulate systems of metallic/non-metallic origin [1,2]. Nanoscale drug-delivery systems, such as vesicular systems (like liposomes, ethosomes, niosomes, and nanoemulsion), and nanoparticulate systems (like polymeric NPs, lipid-based NPs, and inorganic NPs) have been investigated to enhance the topical delivery of different therapies in various skin disorders including psoriasis [34]. The subsequent section discusses the various nanoscale drug-delivery (nanotechnology-mediated drug delivery) approaches utilized for the topical administration of anti-psoriatic drugs. 

### 5.1. Liposome

Out of the various available drug-delivery systems, liposomes have appeared as one of the promising drug-carrier systems for the topical administration of anti-psoriatic agents. It consists of a phospholipid bilayer surrounding an aqueous core [84]. The phospholipid bilayers of liposomes have a similar arrangement of the cellular membrane lipid composition which permits the penetration of loaded therapeutics into the epidermal skin barrier to a better extent [85].

Psoralen in combination with ultra-violate A radiation (PUVA) is an FDA-approved therapy regimen for the treatment of a severe form of recalcitrant psoriasis [86]. Doppalapudi et al. developed a psoralen-encapsulated liposomal system to improve topical efficacy and safety [86]. The designed liposomal system showed a five-fold increase in skin permeation of the psoralen liposomal system compared to the psoralen solution. The in vivo evaluation of the formulation showed a reduction in the psoriatic symptoms as well as the level of inflammatory mediators (such as TNF-α, IL-22, and IL-17) in the imiquimod-induced experimental psoriatic plaque model. In another study, Zhang et al. carried out a comparative investigation between psoralen-loaded ethosomes and liposomes for topical application in psoriasis [87]. The in vitro skin permeability profile suggested that the biopharmaceutical performance of psoralen-loaded ethosomes was better compared to the psoralen-loaded liposomes. In addition, the psoralen-loaded ethosomes exhibited better biocompatibility with human embryonic skin fibroblasts [87]. Desmet et al. investigated ‘DDC642’ as a novel liposomal carrier, capable of acting on the epidermis of impaired and intact human skin by delivering siRNA molecules, without affecting the blood capillaries or dermis. The DDC642 delivering siRNA was also tested on a psoriasis tissue model which led to the down-regulation of the human beta-defensin 2 psoriasis marker [88]. The topical antipsoriatic efficacy of mIL-4 using ultra deformable cationic liposome (UCL) applied to the K14-VEGF transgenic mouse model was reported by Li et al.; the mice treated with the above analogue displayed a mild psoriasis phenotype [89]. Furthermore, the immune histochemical analysis revealed suppression of hyperplastic and inflamed vessels [89].

Bacterial infection is one of the potential causes of the progress of chronic plaque psoriasis [84]. In this regard, Wadhwa et al. reported the formulation and evaluation of fusidic acid (FA), a steroidal antibiotic liposomal formulation. The imaging study suggested considerable cellular uptake of liposomes (loaded with fluorescent dye) [84]. Further, an in vivo study suggested better availability of the drug at a target site and improvement in the therapeutic efficacy of FA liposomal formulation in comparison to conventional formulation of FA [84]. Deformable liposomes of methotrexate entrapped in oleic acid had shown enhanced skin permeability across epidermis and dermis layers of porcine skin and exhibited a higher flux of methotrexate [90,91]. Similarly, Dubet et al. investigated the use of ethosomal nanovesicles for the transdermal delivery of methotrexate. The in vitro studies showed enhanced skin permeation and were supported by an in vivo imaging study using Rhodamine red dye [92]. The retentive nature of the formulations is also seen through some visual penetration pathways and corneocyte swelling. This highlighted the enhanced penetration profile of ethosomes through vesicle skin interaction studies [92]. Erdogan et al. investigated the delivery of liposomes-loaded tacrolimus incorporated into lotion for immune-mediated skin disorders. The radiotracer studies in a murine model showed a nine times higher concentration of tacrolimus released from the liposomal formulation at the target site compared to the systemic administration [93]. Agarwal et al. studied the liposomal/niosomal vesicular system of dithranol, an anti-psoriatic agent. The in vitro permeation studies performed on mouse abdominal skin showed a significantly improved permeation from the liposomal formulation [94,95]. Knudsen et al. investigated liposomal formulations of calcipotriol. The liposome was fabricated using lipo-polymer poly(ethylene glycol)-distearoyl phosphoethanolamine (PEG-DSPE). The study was performed to examine the protective effect of liposome stabilization on the physicochemical properties and penetration ability. The results showed a correlation between the size of the liposomes and the lipid components and the penetration ability. The smaller the unilamellar vesicles the higher the penetration rate. This suggests that the intact liposomes may penetrate through the skin with respect to the size of the vesicles. Calcipotriol showed a deeper penetration compared to the lipid components suggesting a release of a small amount of the drug from the liposomal formulation during the skin migration [96]. Jain et al. reported the topical delivery of thymoquinone through the liposphere for psoriasis treatment [97]. In vitro studies exhibited a reduction in nitric oxide level and inflammatory cytokines (TNF-α, IL-1β, IL-2, IL-6); whereas, in vivo studies supported a decreased level of TNF-α and IL-17 in psoriatic skin indicating improved efficacy in psoriasis [97]. Walunj et al. formulated cationic liposomal nanocarriers of cyclosporine (Cs) using cholesterol and 1, 2-dioleoyl-3-trimethylammonium-propane (Cs liposomal gels) for topical application and evaluated it in an imiquimod-induced experimental model of the psoriatic plaque. The reduction in psoriasis symptoms and levels of psoriatic cytokines (such as TNF-α, IL-17, and IL-22) were reduced after the topical application of the developed formulation system in the psoriatic plaque model [98]. Wang et al. successfully prepared flexible liposomes co-loaded with transretinoic acid and betamethasone and found that application in gel form can reduce the thickness of the epidermis, alleviating the symptoms of psoriasis by reducing TNF-α and IL-6 levels [99]. Saka et al. prepared a liposome gel formulation of bexarotene (retinoid X receptor agonist, and approved for anticancer activity) and the anti-psoriatic effect was evaluated in an imiquimod-induced experimental model of psoriasis in BALB/c mice. The outcome of the results suggested a reduction in scaling and inflammation including recovery from psoriasis without posing any toxic effects [100]. Further, cytokine level and histopathological analysis supported the outcomes. 

Tacrolimus, an immunosuppressive agent and 10-fold more potent than cyclosporine A (CsA), has been suggested for the management of psoriasis; however, poor cutaneous bioavailability limits its use. Jindal et al. proposed the incorporation of tacrolimus in a lipid nanocarrier system, loading them in a hydrogel base for topical delivery [101]. Therefore, the proposed nanoformulation would efficiently target skin tissues and mitigate psoriatic symptoms with minimal adverse effects. Thus, such a hypothesized strategy would be considered a valued addition to the current psoriasis management strategies [101].

#### Clinical Investigations on Liposome-Based Pharmacotherapy

**Figure 5:**
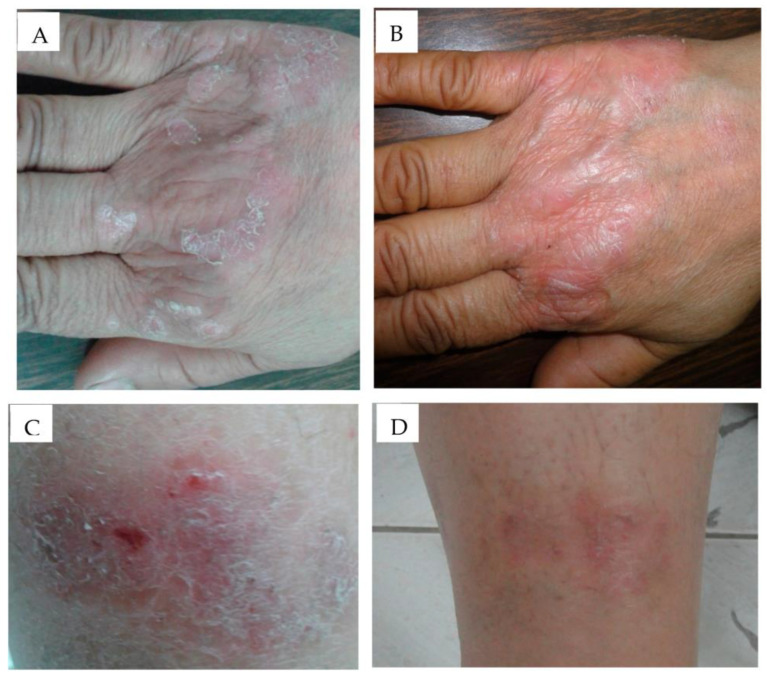
Application of anthralin-loaded liposomal gel (**A**) before application and (**B**) after 3 weeks application and ethosomal gel formulation, (**C**) before application, (**D**) after 3 weeks application has shown significant improvement in psoriatic signs and symptoms in a clinical investigation. Reproduced from [104], MDPI, 2020.

The efficacy of topical CsA in the form of lipogel was assessed in limited chronic plaque psoriasis by Kumar et al. in a single-center randomized clinical trial with 38 patients [102]. The first set consists of 24 patients treated with either cyclosporine lipogel (2.0% *w*/*w*), or placebo lipogel in a randomized way [102]. The second set comprised seven patients treated with cyclosporine lipogel (2.0% *w*/*w*), or conventional cyclosporine cream (2.0% *w*/*w*). In the third set, seven patients were randomly administered cyclosporine lipogel (2.0% *w*/*w*) or clobetasol propionate cream (0.05% *w*/*w*). Patients were assessed for dermatological sum score (DSS) twice weekly for 14 weeks, or until total clearance was observed. The dermatological sum score showed a decrease of 19% in 59% of psoriasis sites after 2 weeks of treatment with cyclosporine lipogel [102]. After eight weeks, a prominent decrease (nearly 83%) was recorded on all sites treated with cyclosporine lipogel [102]. A randomized, double-blind, study was performed by using a solvent-controlled novel liposomal formulation of dithranol in psoriasis by Saraswat et al. [103]. Around twenty patients were selected with symmetrical stable bilateral plaque psoriasis and treated with 0.5% dithranol lipogel to lesions on one side of the body [103]. On the other hand, around ten patients were treated with liposomal base randomly and further ten patients were treated with a conventional cream (containing 1.15% salicylic acid, 1.15% dithranol, and 5.3% coal tar) for 6 weeks period with a short contact regimen of 30 min [103]. Patients were assessed at baseline, 2, 4, and 6 weeks for disease severity, by observing perilesional erythema and skin staining, pruritus, and possible side effects. The clinical investigation revealed that patients treated with lipogel formulation exhibited a markedly low incidence of skin staining and perilesional erythema compared to conventional cream [103]. Agarwal et al. reported preliminary observations of a novel liposomal formulation containing dithranol in treating stable plaque psoriasis. The formulations were evaluated for their efficacy, tolerability, and cosmetic acceptability [95]. In a prospective, open-label trial, nine patients with nineteen psoriatic plaques were treated for six weeks. In this clinical study, five patients showed no lesions or perilesional irritation with complete lesion clearance, one patient exhibited more than 50% clearance and only one patient showed faint brown skin staining, which was completely reversed quickly [95]. Recently Fathalla et al. designed and developed an anthralin-loaded liposomal gel and ethosomal gel formulation to evaluate its clinical efficacy in 44-year-old male patients [104]. It was observed that both the developed formulations were helpful to minimize the psoriatic sign and symptoms after 3 weeks of treatment very effectively as shown in Figure 5. 

### 5.2. Niosomes

Niosomes are non-ionic surfactant-based unilamellar or multilamellar vesicular drug-delivery systems containing a central compartment of an aqueous solution of solute surrounded by a bilayer membrane resulting from the organization of surfactant macromolecules [105]. Niosomes are considered to be a promising system for the topical delivery of therapeutics due to their biocompatibility, non-immunogenicity, and other outstanding features such as enhanced drug penetration abilities, sustained drug release, improved drug stability and capabilities to carry lipophilic as a well hydrophilic drug [106]. 

Moghddam et al. developed the diacerein-entrapped niosomes formulated by the incorporation of cholesterol for the management of psoriasis [107]. The confocal laser scanning microscopic studies revealed an enhanced penetration of diacerein in the epidermal and dermal skin layers of rats [107]. This study provides a way for targeting and enhanced drug delivery through the topical application of niosomes which, in turn, reduces the adverse effects and toxicity associated with systemic administration [107]. In another study, the formulation of nanosponge and niosomes incorporated gel (NSG and NMG, respectively) containing tazarotene was demonstrated by Aggarwal et al. [108]. Their results revealed that NSG and NMG gel formulations reduce the side effects associated with conventional gel formulations and marketed formulations and improve the bioavailability of tazarotene. The prepared NSG and NMG gel formulations showed increased skin retention and local accumulation efficiency (LAE) [108]. Gupta et al. investigated the protective effect of capsaicin (CAP)-loaded niosomes, liposomes, and emulsomes for the controlled local delivery of drugs for psoriasis treatment [109]. The in vitro and in vivo studies showed prominent enhancement in skin retention of CAP from emulgel formulation [109]. Finally, their results suggested that emulgel is a plausible approach for the topical application of CAP, to achieve an effective treatment for psoriasis [109]. Parnami et al. explored the antipsoriatic activity of methotrexate and CsA in niosomal formulations for antipsoriatic activity associated with narrow and broad-band UV radiation [110]. The prepared formulations exhibited increased drug accumulation and decreased adverse effects associated with systemic delivery [110]. Bracke et al. explored the formulation of DEFB4-siRNA-containing SECosomes for topical delivery. The targeted delivery to Human β defensin-2 (hBD-2) is achieved by using a bioengineered skin-humanized mouse model for psoriasis which showed an improved psoriatic phenotype leading to normal skin structure [111]. The study revealed a filaggrin expression with the retrieval of trans-glutaminase activity and recovery of stratum corneum similar to normal human skin regeneration [111]. Bhatia et al. formulated the Tamoxifen (TAM) loaded flexible membrane vesicles (FMVs) and pluronic lecithinized organogels (PLOs) [112]. The antipsoriatic studies performed on mice tail models showed significantly higher efficacy of TAM-FMV gel and TAM-PLO compare to the conventional TAM-hydrogel [112]. Ammonium glycyrrhizinate niosomes as a potential nano-vesicular system for psoriasis treatment employing murine models was proposed by Marianecci et al. [113]. Their results suggested that drug-loaded niosomes shows reduced edema and nociception in comparison to placebo niosomes and drug alone [113]. Meng et al. developed a novel topical niosomes loaded with celastrol and, through an in vivo study, suggested its efficacy in alleviating psoriasis symptoms (erythema and scaling) in a mouse model of psoriasis [114]. Further, spleen size and the cytokines levels (IL-22, IL-23, and IL-17) were also decreased after noisome treatment, supporting their promising therapeutic prospects in psoriasis [114]. Al-Mahallawi et al. incorporate methotrexate (MTX) into ultra-permeable niosomal vesicles and their optimized formula demonstrated improved drug permeation and a significant deposition of MTX in rat dorsal skin, while histopathological evaluation confirmed the tolerability of the optimized formula in rats upon topical application [115]. 

#### Clinical Investigations on Niosomes-Based Pharmacotherapy

Lakshmi et al. conducted a double-blind placebo-controlled study and accessed the efficacy of niosomal MTX in chitosan gel on psoriasis patients [116]. Skin irritation and sensitivity are tested by the insult patch test (HRIPT) [116]. The psoriasis lesion area and severity index scoring method was used to calculate the efficacy. The niosomal methotrexate gel formulations exhibited an enhanced efficacy in comparison to placebo and marketed gel formulations [116]. Moreover, a similar approach to demonstrate the efficacy of a novel sustained release niosomal chitosan-MTX formulation in comparison to placebo gel and a plain MTX gel for different types of psoriasis treatment, especially palmoplantar psoriasis was attributed by Lakshmi et al. [117]. The treatment results showed a marked improvement in the lesions treated with niosomal chitosan-MTX formulation in comparison to plain MTX and placebo gel even though applied twice a day. The study suggests that the novel formulation did not exert any systemic toxicity and showed improved therapeutic potential without altering biochemical parameters [117]. The various preclinical/clinical investigations utilized lipid-based vesicular systems (such as liposome, ethosome, and noisome) to improve the biopharmaceutical attributes/therapeutic efficacy of encapsulated drugs/actives in the management of psoriasis are summarized in Table 3. 

### 5.3. Solid Lipid Nanoparticles

Solid lipid nanoparticles (SLN) have materialized as a substitute for liposomal drug-delivery systems owing to their several advantages such as enhanced physical stability, reasonably inexpensive compared to phospholipids, and simple manufacturing technique [121,122,123]. Furthermore, their potential in controlled drug delivery [121,122], topical drug delivery in epidermal targeting [124,125], and stability of active pharmaceutical ingredients have been very well established and are more or equivalent to liposomes [121]. An SLNs-loaded topical gel containing Betamethasone dipropionate and Calcipotriol delayed the abrupt growth of keratinocytes in the HaCaT cell line [126]. Furthermore, in comparison to the marketed Daivobet^®^ ointment, the formulated SLN gel exhibited reduced epidermal depth and improved melanocyte count in an experimental mouse tail model of psoriasis [126]. Bikkad et al. reported the preparation and characterization of Halobetasol propionate (HP)-loaded SLNs gel and improved drug deposition avoiding systemic uptake and acting as a probable carrier for local delivery with reduced side effects. The ex vivo studies showed continued drug release for 12 h and the in vitro drug deposition study exhibited that HP-SLN formulations exhibited better drug accumulation [127]. Moreover, the skin irritation studies of HP loaded SLNs gel suggested better safety and tolerability as compared to commercial products [127]. Similarly, Madan et al. reported that SLNs loaded with Mometasone furoate are a potential tool for skin targeting over the conventional formulations of corticosteroid drugs [128]. Triamcinolone acetonide (TA) loaded SLNs presented extended drug release following Higuchi release kinetics. Skin distribution studies of TA-loaded SLNs demonstrated improved drug accumulation without its systemic uptake [129]. Agarwal et al. demonstrated the ability of SLN and nanostructured lipid carriers (NLCs) for skin targeting capsaicin. NLCs and SLNs proved to be potential carriers for improved local delivery exhibiting better drug accumulation in different layers of the skin [130]. SLNs loaded with dithranol were formulated by Gambhire et al. They reported that the incorporation of dithranol in SLNs reduced the rate of drug degradation [131]. Kim et al. demonstrated SLN loaded with Cyclosporin A (CsA) [132]. In vitro permeation studies of CsA-SLN showed a two-fold higher skin permeation efficiency compared to a CsA-oil mixture in viable skin [132]. Moreover, the in vivo studies in a mouse model exhibited reduced symptoms of atopic dermatitis by decreasing cytokines interleukin (IL)-4 and -5 of T helper (Th) 2 cells [132]. Shah et al. investigated the viability of tretinoin (TRE) loaded SLN gel for improved transdermal delivery of TRE [121]. TRE-loaded SLN showed significantly improved photostability [121]. Furthermore, it was also reported that SLN-based TRE gel showed decreased skin irritation compared to commercial TRE cream [121]. Similarly, Ridolfi et al. reported TRE-loaded chitosan-SLN loaded to provide a dual effect which is the anti-bacterial activity of chitosan combined with the effect of TRE for topical treatment of psoriasis [133]. The local delivery of isotretinoin from SLN (IT-SLN) was evaluated by Liu et al. [125]. The in vitro permeation studies showed no systemic uptake of isotretinoin in skins and augmented skin targeting effect by all the IT-SLN loaded formulations [125]. Trombino et al. prepared SLN, based on trehalose monooleate, loaded with cyclosporin-A for possible management of psoriasis [134]. The optimized formulation indicates the possibility of using such nanocarriers of cyclosporin-A for topical application in psoriasis, diminishing possible adverse effects due to the expected systemic translocation of cyclosporin-A and, meanwhile, augmenting its concentration at skin lesions [134].

### 5.4. Nanostructured Lipid Carrier

NLCs are modified unstructured-matric of SLN, i.e., consist of both solid and liquid lipid as a core matrix [135,136]. The assimilation of liquid lipids with a solid matrix of NPs facilitates the incorporation of an increased amount of drug and results in enhanced solubility, improved stability, increased permeability, and bioavailability, and most important the prolonged t_1/2_ and targeted drug delivery [135,136,137,138,139,140]. Furthermore, close contact of NLCs with stratum corneum and smaller particle size results in enhanced drug flux through the skin. On the other hand, the presence of solidified lipid matrix results in the controlled release of therapeutic ingredients [135,136,137,138,139,140,141]. Moreover, it has been also reported that NLCs exhibit occlusive properties and significantly increase skin hydration, and minimize transdermal water loss [141]. 

Agarwal et al. reported the formulation and evaluation of Acitretin-loaded NLCs. The prepared formulations exhibited higher skin deposition compared to plain acitretin gel in human cadaver skin. Moreover, the better efficacy and reduced local side effects exhibited by acitretin NLCs loaded gel in clinical studies make it a potential carrier for the topical administration of drugs in psoriasis [142]. Pinto et al. reported MTX loaded NLCs show higher skin penetration in comparison to free MTX, this signifies the role of nanocarriers in topical drug delivery [143]. Similarly, MTX-loaded NLCs (MTX-NLC) nanogel formulations showed a decreased PASI score recovering mice’s skin to normal level [144]. The combined effect of calcipotriol with MTX significantly strengthens the topical treatment of psoriasis [145]. Lin et al. proposed NLCs loaded with both lipophilic and hydrophilic drugs to demonstrate the topical delivery of Calcipotriol and MTX, respectively [145]. The skin permeation studies of NLCs loaded with dual drugs exhibited decreased skin penetration by calcipotriol in comparison to MTX [145]. The results of in vitro and in vivo studies examined by confocal laser scanning microscopy (CLSM) exhibited a good correlation [145]. Dual drug-loaded NLCs showed improved drug permeation and decreased skin irritation making it a suitable carrier system for the local administration of antipsoriatic drugs [145]. Tripathi et al. investigated and reported the influence of the carrier system on drug release and drug deposition efficiency through a carbomer gel containing MTX-loaded NLC and MTX-loaded SLN for topical administration of MTX in the management of psoriasis [146]. The skin permeation studies showed extended drug release of up to 24 h from both the carrier systems loaded with MTX through hydrogels [146]. However, among the two carrier systems, NLC hydrogels exhibited an improved skin drug deposition (28.8%) compare to SLN hydrogel (18.6%) and plain drug-loaded hydrogel (11.4%) [146]. A modified nano lipid carrier (MNLC) for topical delivery of tacrolimus was developed by Pople et al. [147]. Tacrolimus-loaded MNLC (T-MNLC) gels exhibited significantly enhanced skin penetration, and bioavailability in comparison to marketed preparation [147]. Furthermore, skin irritation studies also showed a reduced level of skin irritation from T-MNLC compared to marketed products [147]. Similarly, Thapa et al. reported the formulation and characterization of Tacrolimus-loaded liquid crystalline nanoparticles. The liquid crystalline nanoparticles resulted in extended drug release for up to two weeks and showed enhanced storage stability of Tacrolimus from nanoparticles [148]. Pradhan et al. demonstrated the Fluocinolone acetonide (FA) loaded NLCs for improvement in skin permeation efficiency of the drug in the treatment of psoriasis. In vitro skin distribution studies from NLCs and a plain suspension containing FA showed relatively better drug penetration NLCs in the skin layers [36]. The skin permeating potential of polymeric micelles loaded with TAC was demonstrated by Lapteva et al. The skin deposition studies revealed enhanced drug permeation and deposition into dermal/epidermal layers through polymeric micelles compared to conventional ointment [149]. The cutaneous distribution profile of TAC exhibited an enhanced level of TAC in the upper layer of the skin due to augmented TAC deposition in the stratum corneum, with no increased deposition in the lower deeper layers of the skin [149]. The permeation studies revealed no deeper skin deposition of the drug; thus, site targeting can be achieved with decreased adverse effects [149]. Furthermore, CLSM of the fair follicles also showed drug deposition in the follicles acting as a potential system for skin targeting with no systemic uptake and reduced side effects [149]. The first time the synergic combination was reported was by Viegas et al. by formulating a multifunctional NLC to co-administer tacrolimus and TNF-α siRNA for psoriasis treatment by targeting TNF-α [150]. The outcome of the experimental study demonstrated the efficiency of the multifunctional NLC, by reducing TNF-α expression about seven-fold and exhibiting the synergic effect of tacrolimus and TNF-α siRNA [150]. The developed system was successful in limiting the symptoms of disease in an imiquimod-induced experimental model of psoriasis. NLCs loaded with curcumin and caffeine were formulated by Iriventi et al. through a hot homogenization and ultrasonication approach and added to topical gel have provided a continued release of the drug up to 12 h [151]. An in vivo efficacy study was carried out in an imiquimod-induced mouse model of psoriasis [151] and the resulting outcome suggested improved efficacy of curcumin and caffeine in psoriasis [151]. Qadir et al. developed an NLC gel of two potential herbal agents, Smilax china and Salix alba, for safe and effective topical therapy against psoriasis [152]. During the in vivo study, they expressed a non-irritant profile of the developed system and better PASI score, and better therapeutic efficacy as compared to the marketed preparation [152]. Sharma et al. develop Squalene integrated NLC gel of tamoxifen citrate to enhance the lipid and water content of skin and to create a local depot in the skin for better efficacy in psoriasis. The outcome of the in vivo study suggested better efficacy in dampening PASI scores, pro-inflammatory cytokines level, and reinstating splenomegaly and other histological alterations to normal [153]. The clinical pertinence of this stable gel ensured its longer stay (owing to semisolid viscosity) over the skin, augmented skin hydration (due to hydrophilic gelling polymer) and lipid content (owing to the natural skin lipid composition), and better skin retention of drug and overall better efficacy in psoriasis [153]. Rapalli et al. prepared curcumin-loaded NLC to improve the penetration of curcumin (poorly soluble) onto a topical layer of skin for the management of psoriasis and *Acne vulgaris* [154]. An in vitro release study of Curcumin-NLC suggested an extended-release profile up to 48 h; whereas, plain curcumin released 100% drug within 4 h. Further, the ex-vivo permeation study of curcumin NLC gel exhibited more than three-fold higher drug permeation and retention in comparison to free curcumin gel [154]. The various preclinical investigations utilized lipid-based nanoparticulate systems (SLN, NLC) to improve the biopharmaceutical attributes/therapeutic efficacy of encapsulated drugs/actives in the management of psoriasis are summarized in Table 4. 

### 5.5. Polymeric Nanoparticles

In the last few decades, polymeric nanoparticles (PNPs) have attracted the great attention of formulation scientists because of their unique properties and performance due to their small size [156,157,158]. PNPs with readily tunable physicochemical properties can effectively stabilize unstable drugs, minimize their adverse effect, control the release of drugs, and potentiate the penetration of drugs across the skin barrier [159]. 

The therapeutic potential of Dead Sea Water (DSW) for skin diseases s have been well established [160]. DSW enriched with minerals (Ca, Mg, Na, K, Zn, and Sr) are known to utilize anti-inflammatory effects and enhance skin barrier recovery [160]. The formulation and evaluation of DSW-based PNPs were reported by Dessy et al. The resulting NPs show a time-regulated drug release and a better formulation yield [160]. Mao et al. designed the skin-permeating PNPs in a silk fibroin hydrogel-based matrix to simplify the administration of curcumin deeper in skin layers. Curcumin-NPs incorporated in silk fibroin hydrogel (CUR-NPs-gel) exhibited a steady release of curcumin as compared to CUR-gel, without affecting the skin penetration ability of CUR-NPs [161]. In in vivo studies, using an imiquimod-induced psoriatic mice model, treatment with CUR-NPs-gel exhibited a better therapeutic profile than CUR-NPs which previously demonstrated greater skin-permeation profile and efficacious in the anti-keratinization process [161]. Thus, the CUR-NPs-gel resulted in the inhibition of inflammatory cytokines expression (TNF-α, NF-κB, and IL-6) [161]. Similarly, Bilia et al. reported that CUR-PNPs potentiate the protective efficacy to act in moderate-to-severe psoriasis patients and regulate serum cholesterol levels [162]. Shah et al. developed an effective skin permeating nanogel system (SPN) for the cutaneous co-delivery of two anti-inflammatory drugs (spantide II: SP; and ketoprofen: KP) [163]. An in vitro permeation study exhibited enhanced accumulation of SP for the SP-SPN or SP+KP-SPN in the epidermis was better than SP-gel [163]. Further, the accumulation of KP for KP-SPN or SP+KP-SPN in the epidermis and dermis was better than KP-gel [163]. Simultaneously, KP permeation in KP-SPN or SP+KP-SPN was enhanced more than nine-fold in comparison to KP-gel. The thickness of the ear in the ACD model, PASI score, and IL-17 and IL-23 expression were significantly attenuated in SPN treatment in comparison to normal gel [163]. Pukale et al. reported a scalable and stable monolithic lipid–polymer hybrid nanoparticles (LPNs) of clobetasol propionate (CP) consisting of a combination of solid/liquid lipids along with amphiphilic copolymer, mPEG-PLA [164]. The prepared nanoparticles were encapsulated in the hydrophobic core of LPNs and the clobetasol-loaded LPNs (CP/LPNs) were prepared in a dermal hydrogel using carbopol 974P [164]. CP/LPNs gel exhibited a controlled in vitro drug release for 7 days with no abrupt release [164]. Further, topical administration of CP/LPNs gel on an experimental model of psoriasis-like inflammation exhibited minimal systemic penetration of clobetasol [164]. An in vivo efficacy assessment study of CP/LPNs gel resulted in prominent improvement in PASI score, and attenuation of skin damage in comparison to commercialized products (Clobetamos™) [164]. Overall, improved cellular uptake, better skin penetration with augmented dermal retention, and improved therapeutic efficacy demonstrate the promising features of these LPNs for further clinical investigations [164]. The topical delivery of dithranol through dendritic nanocarriers was reported by Agarwal et al. [165]. The polypropylene imine (PPI) dendrimers for topical administration of dithranol (DIT) were evaluated for skin deposition, encapsulation efficiency, and skin irritation [165]. The studies exhibited significantly improved permeation rate constant and decreased skin irritation with DIT-PPI compare to plain DIT [165]. The biodistribution studies of dye-loaded dendrimers revealed by confocal laser scanning microscope images exhibited enhanced drug accumulation in the epidermal and dermal layers posing it as a potential carrier system for skin targeting for psoriasis treatment [165]. The various preclinical investigations utilized polymeric nanoparticulate systems to improve the biopharmaceutical attributes/therapeutic efficacy of encapsulated drugs/actives in the management of psoriasis are summarized in Table 5. 

### 5.6. Nanoemulsion/Microemulsion-Based System

Nanoemulgel (NEG) is an assimilated formulation of two diverse systems in which drug-loaded nanoemulsion (NE) is incorporated into a gel base [172]. NEG has been established as a promising alternative drug-delivery option with enhanced permeability and bioavailability of lipophilic drugs [173]. The amalgamation of the two different systems makes it beneficial in numerous ways, such as easy incorporation of lipophilic drugs and enhanced skin permeability of incorporated drugs [172], an enhanced retention time of drug at the targeted area, and conclusively, results in maximum therapeutic potential with less side effect [174,175]. 

Divya et al. reported the formulation and evaluation of topical NEG formulation of two anti-psoriatic drugs (acitretin: Act and Aloe-emodin: AE) using chitin [176]. The ex vivo study suggested enhanced dermal drug deposition [176]. The in vivo anti-psoriatic study, and skin safety study revealed the plausible therapeutic benefit of topical administration of Act and AE in psoriasis [176]. Ourique et al. reported the preparation of TRE-loaded LPN in hydrogels, which demonstrated a lower photodegradation than non-encapsulated drugs [177]. The lag time was augmented two-fold with nanoencapsulation, indicating enhanced drug retention on the skin surface [177]. The development and optimization of CP and calcipotriol (CT) NEG for the topical management of psoriasis were reported by Kaur et al. [178]. An in vitro study, using HaCaT cell lines, elicited a better uptake of the drug with enhanced penetration in stratum corneum and viable layers from NEG as compared to free drugs [178]. Imiquimod-induced experimental psoriatic BALB/c mice model suggested an improved anti-psoriatic effect of NEG in comparison to free drugs or commercialized products [178]. A comparative therapeutic efficacy study of clobetasol-loaded chitin nanogel (CLCNEG) and marketed cream were performed by Panonnummal et al. [179] and a significant anti-inflammatory effect was observed via inhibition of COX and LOX activities. Further, an in vivo anti-psoriatic study using an imiquimod-induced experimental model of psoriasis revealed the potential therapeutic benefits of NEG through topical administration of clobetasol in psoriasis [179]. Similarly, Alam et al. carried out an in vivo study of clobetasol propionate-loaded nanoemulsion through topical treatment in psoriasis and atopic dermatitis [180]. The clobetasol propionate-loaded nanoemulsion significantly elevated lymphocytic nucleoside triphosphate phosphohydrolase activity, an enzyme responsible for extracellular ATP degradation and responsible for cell differentiation, proliferation, and inflammatory cascade [180]. Baboota et al. investigated the potential of the nanocarrier-based hydrogel of betamethasone dipropionate and salicylic acid for the management of psoriasis [181]. They reported enhanced drug deposition beneath the skin and improved anti-inflammatory activity against psoriasis [181]. MTX-loaded chitin nanogel (MCNEGs) in the imiquimod-induced experimental psoriatic model resulted in significant anti-psoriatic efficacy without any dermal and systemic toxicity [182]. MCNEGs have shown prominent anti-inflammatory properties via inhibition of COX-2 and LOX-5 enzyme expression in THP-1 cells [182]. Similarly improved topical delivery and anti-inflammatory activity of MTX from a nanogel were studied by Singka et al. They reported that the increased concentration gradient, high flux, and reduced prostaglandin E (2) (PGE_2_) synthesis is in an ex vivo study [183]. Curcumin-loaded PLGA nanoparticles (Cur-NPs) in hydrogel were prepared and delivered topically to treat imiquimod-induced psoriasis [184]. In vitro skin permeation studies revealed better drug permeation through the skin when administered as the Cur-NPs-loaded hydrogel in comparison to normal drug suspension-loaded hydrogel [184]. Similarly, Cur-NPs-loaded hydrogel showed improved anti-proliferative activity than Cur solution in the cell line study [184]. Khurana et al. prepared resveratrol-loaded polymeric micelles (PM) and converted them into gel (PMG) and in vivo efficacy was evaluated in an imiquimod-induced psoriatic-like plaque model [185]. The in vivo study represented the prominent efficacy of PMG (as apparent through reduced PASI score, cytokines levels, splenomegaly, and hyperkeratosis) in comparison to conventional gel [185]. Li et al. formulated a cyclosporine-loaded Pluronic^®^ F127 stabilized reduced graphene oxide hydrogel to enhance permeation and retention of cyclosporine in the skin for better therapeutic efficacy in psoriasis [186]. They reported the ability of the reduced graphene oxide nanocarrier to the betterment of cyclosporine permeation, and retention in dermal layers could be used for better psoriasis management, with minimal adverse effects observed through oral/systemic routes [186]. Song et al. loaded calcipotriol in exo-polysaccharides/calcipotriol (EPS/CPT) emulsion, sunflower oil, and calcipotriol as the loaded drug, and the therapeutic effect was evaluated [187]. The in vitro and in vivo studies expressed better efficacy for the management of psoriasis vulgaris by increasing the aggregation of CPT in psoriatic lesions and attenuating pro-inflammatory cytokine levels [187].

**Figure 6:**
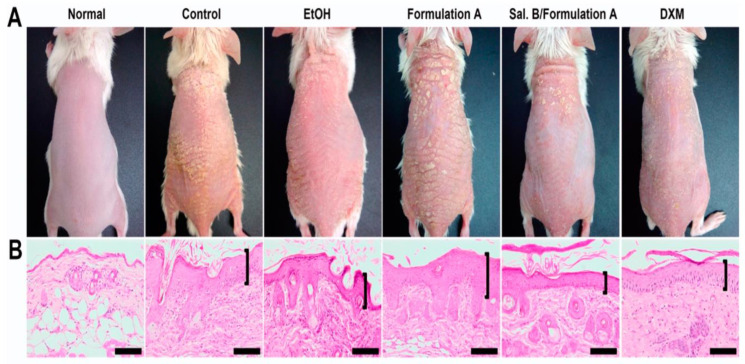
(**A**) Salvianolic acid B loaded microemulsion system (Sal. B/formulation A) and 0.25% desoximetasone marketed ointment (DXM) improved the sign and symptoms in psoriasis-like dermatitis compared to another treatment group (control group: IMQ-induced psoriatic mice without any treatment; EtOH group: treatment with ethanol in IMQ-induced psoriatic mice; formulation A group: treatment with microemulsion base/vehicle only in IMQ-induced psoriatic mice) after 6 days of treatment. (**B**) The Sal. B/formulation A and DXM treatment groups exhibited fewer severe clinical and pathological features (epidermal acanthosis, parakeratosis, tortuous capillary dilatation in papillary dermis, and inflammatory cell infiltration) compared to other groups (control group; EtOH group; formulation A group) in a histopathological investigation. Scale bar = 100 μm. Reproduced from [191], MDPI, 2020.

Shinde et al. aimed to develop the mometasone furoate vesicle containing aspasomal gel for the management of psoriasis [188]. The prepared gel exhibited a longer duration of drug release and thus required a lower dosing frequency. Therefore, such approaches are capable of providing a rational future approach for the management of psoriasis [188]. Dadwal et al. prepared clobetasol propionate-loaded squarticles (nanoemulgel) for the management of psoriasis by providing better skin permeation and enhancing its therapeutic efficacy [189]. Erol et al. prepared tazarotene-loaded in situ gels using thermosensitive poloxamers via the cold method for the potential management of psoriasis [190]. The in vitro study, using LPS-stimulated RAW264.7 murine macrophage cells suggested a reduction in nitric oxide and PGE_2_ levels and supported its anti-inflammatory and analgesic activities. The 10% tazarotene in situ gels have shown better efficacy. Thus, it may act as a potentially safer alternative for tazarotene-based topical formulation for the management of psoriasis [190]. Recently, Guo et al. designed and developed a Salvianolic acid B-loaded microemulsion system to evaluate its preclinical efficacy in imiquimod-induced psoriasis-like dermatitis in mice models [191]. It was observed that the developed formulations were helpful to minimize the sign and symptoms of psoriasis-like dermatitis in mice after 6 days of treatment very effectively as shown in Figure 6. 

### 5.7. Metallic Nanoparticles

Metallic nanoparticles (MNPs) have fascinated researchers for over a decade and are now greatly utilized for biomedical applications [192]. MNPs usually have wide utility in biomedical applications including drug-delivery vehicles in various disease conditions with good biocompatibility, affluent functionality, and controlled release of therapeutic agents [192,193]. In recent years, two-dimensional (2D) materials of metallic origin like nanosheets are also employed as a smart drug-delivery system in different disease conditions [194,195]. It has been reported that MNPs show great advantages not only as anti-aging and anti-acne treatments, and skincare products but also for the management of various skin disorders including psoriasis [196]. 

Gold nanoparticles (GNPs) appeared as a preferred choice for the topical application system of different drugs. Taking into the above consideration Bessar et al. reported the development and evaluation of MTX-loaded water-soluble GNP activated with sodium 3-mercapto-1-propane sulfonate (Au-3MPS) [197]. The results of in vitro studies suggested that MTX conjugated with Au-3MPS is more efficacious than MTX alone. Cerra et al. highlighted the interaction between functionalized GNP and MTX at a molecular level [198]. Monodisperse GNP was prepared by a chemical method using hydrophilic a functionalizing agent (thiol 3-mercapto-1-propane sulfonate) [198]. They suggested such a delivery system would potentiate drug efficacy and help in deciphering pharmaco-dynamic/kinetic properties for the development of effective drug-delivery systems for psoriasis [198]. Recently, Han et al. designed and developed alkyl-terminated GNP as a self-therapeutic system to treat psoriasis [199]. It was observed that the developed system is helpful to minimize the sign and symptoms of imiquimod-induced psoriasis in mice by down-regulating the interleukin-17 signaling pathway. It effectively crosses the stratum corneum and enters into keratinocytes easily due to the small dimension (<15 nm) of the developed system. In another investigation, Fereig et al. designed and developed a self-assembled hybrid system of GNP to treat psoriasis [200]. The developed formulation system was prepared by hybridization of GNP with lecithin-chitosan nanoparticles. It was observed that the developed tacrolimus-loaded system is helpful to minimize the inflammatory markers in the imiquimod-induced mouse model with a significant anti-psoriatic effect. 

In another investigation, silver nanoparticles (AgNPs) using European black elderberry (*Sambucus nigra*, Adoxaceae family) fruit extracts were prepared and assessed for anti-psoriatic activity on HaCaT cells exposed to UVB radiation, on an acute inflammation model [201]. Their results exhibited anti-inflammatory properties by reducing inflammatory cytokines generation through UVB irradiation [201]. Further, Lai et al., in an investigation, explored the risk of applying zinc oxide nanoparticles (ZnONPs) to treat psoriasis [202]. It was observed that ZnONPs delay the recovery of psoriasis-like skin lesions by promoting inflammation and keratinocyte apoptosis via the nuclear translocation of phosphorylated NF-κB p65 and cysteine deficiency. The findings of the current investigation reveal that the application of ZnONPs as a topical drug-delivery vehicle is not desirable for pathological skin, although it has been utilized after percutaneous administration in other disease conditions.

## 6. Targeted Pharmacotherapy in the Management of Psoriasis

Available pharmacotherapy in the management of psoriasis may be categorized into three classes dermal therapy, phototherapy, and systemic therapy. Phototherapy is normally advised in cases when the topical application is not effective. After phototherapy, systemic therapy is prescribed for better results. The limitations of phototherapy and systemic therapy are associated with various prominent adverse effects, including hepatic/nephrotoxicity, hypertension, hyperlipidemia, and skin cancer. The adverse events associated with other than dermal therapy, made topical pharmacotherapy still the preferred therapy in the management of psoriasis. We know that the currently available pharmacotherapy based on conventional dosage forms is associated with frequent dosing, chances of side effects, and the possibility of toxic events due to continuous therapy for a longer duration in the management of psoriasis [1,2].

**Figure 7:**
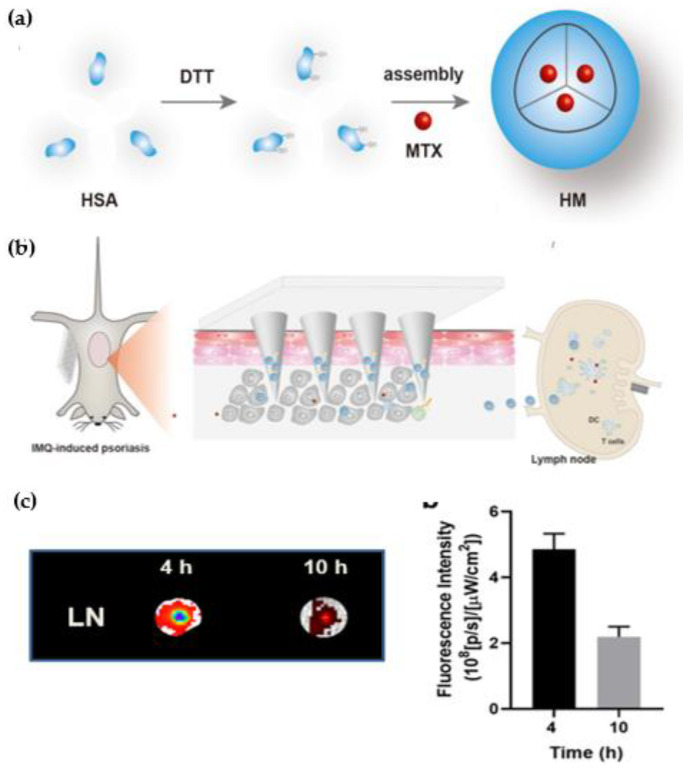
Schematic illustration shows concept of targeted drug delivery in psoriasis. (**a**) Synthesis of hyaluronic acid-based albumin nanoparticles by hydrophobic interactions between human serum albumin and methotrexate therapeutic. (**b**) Delivery of methotrexate utilizing hyaluronic acid-based albumin nanoparticles for effective inhibition of dermatitis. Active targeting of developed formulation system helpful to deliver therapeutics in subcutaneous tissue and further accumulated in lymph nodes suppress the immune cells and prevent the trigger of pro-inflammatory cytokines secretion to improve the progression of psoriasis utilizing microneedle patch delivery system. (**c**) Fluorescence images of lymph nodes shows fluorescent intensities from the lymph nodes after administration of developed targeted formulation system at 4h and 10h. HAS: Human Serum Albumin; MTX: Methotrexate; HM: Hyaluronic-acid based microneedle patch; DC: Dendritic Cells; LN: Lymph nodes. Reproduced from Wang H et al. [171], Dove Medical Press, 2022.

The recent approach of targeted pharmacotherapy therapy in the management of psoriasis has advantages over conventional therapy [203,204]. It appears safe and effective in the delivery of anti-psoriatic drugs and maintains the concentration of the drug locally to better treat psoriasis. If the drug is targeted, the dose requirement is less, which eventually reduces the possibility of systemic adverse effects even though the therapy is topical. In addition, topical applications of drugs have limited release, degradation of drug, and poor efficacy due to the barrier of the stratum corneum layer. Further, the targeted formulation provides a prolonged duration of action, reduction in dosing frequency, and overall improves patient compliance. The targeting of receptors of psoriasis may also be helpful in phototherapy and systemic therapy by molecular recognition, facilitating drug formulation to specifically interact with pathogenic molecular targets [205]. Two approaches (passive and active) are commonly exploited in targeted pharmacotherapy. If the drug is accumulated in a specific site of a targeted area due to its biophysical properties, this type of targeting is called passive targeting. An example of passive targeting is the site-specific delivery of anti-psoriatic therapeutics at nanoscale dimension and their differential distribution in skin region is based on the size of the therapeutics-loaded particles [104]. That is why in this targeting approach, the delivery of the therapeutic system is manipulated at nanoscale dimension employing nanodrug carrier systems (Illustrated in Figure 4 and Figure 5). Active targeting, which is also considered molecular or biological targeting, may utilize physical forces such as electroporation, sonoporation, and magnetism including the conjugation of targeting ligands to target cells to produce therapeutic effects at the disease site. The ligand-mediated targeting can be achieved by fixing the target-specific peptides or target-specific antibodies attached to the drug-delivery system [206,207]. Targeted pharmacotherapy in the management of psoriasis is a promising strategy to deliver the loaded therapeutics to specific cells in the skin, which emerges from a combination of inflammatory, and immune responses in psoriatic skin. The recent investigation highlights the significance of targeted drug delivery of methotrexate utilizing hyaluronic acid-based albumin nanoparticles to deliver the loaded therapeutics in lymph nodes to suppress the immune cells of the acquired immune system in psoriasis through microneedle patch delivery (illustrated in Figure 7) [171]. 

### 6.1. Targeting through Serotonergic Receptors

Various researchers have reported the involvement of various serotonergic receptors in psoriasis [208,209,210,211]. Laberge et al. reported that 5-HT1AR is involved in the inhibition of inflammation [208] while Morita et al. reported the involvement of 5-HT7R in itching [209]. Weisshaar et al. reported the presence of 5-HT_3_R while Nordlind et al. suggested the presence of 5-HT_1A_R and 5-HT_2A_R expression in psoriatic skin [210,211]. The differential expression of 5-HT_1A_ and more 5-HT_2A_ receptors were reported [212] in the structures of psoriatic skin in an immune-histochemical investigation and suggested the possibility of targeting through serotonergic receptors could be a promising approach in the management of psoriasis.

### 6.2. Targeting through Dopamine Receptors

Dopamine receptor D1 (DRD1) has been reported for its anti-proliferative potential by binding to DRD1 on activated T cells and killing by causing the minimal death of resting normal human T-cells [213,214]. Fenoldopam mesylate, 6-chloro-2, 3, 4, 5-tetrahydro-l- (4- hydroxyphenyl)–1H-3-benzazepine-7, 8-diol, methane sulfonate (FD, a highly selective DRD1 agonist) therapeutic efficacy in psoriasis has been suggested by several researchers [86,119,215]. Doppalapudi et al. formulated the water-washable ointment and glycerin-based carbopol anhydrous gel of FD [216]. The developed formulation system was evaluated in the imiquimod-induced psoriasis model and suggested its utility as targeted pharmacotherapy to suppress the inflammatory condition in psoriasis [216].

### 6.3. Targeting through microRNAs (miRNA-Based Targeting)

**Figure 8:**
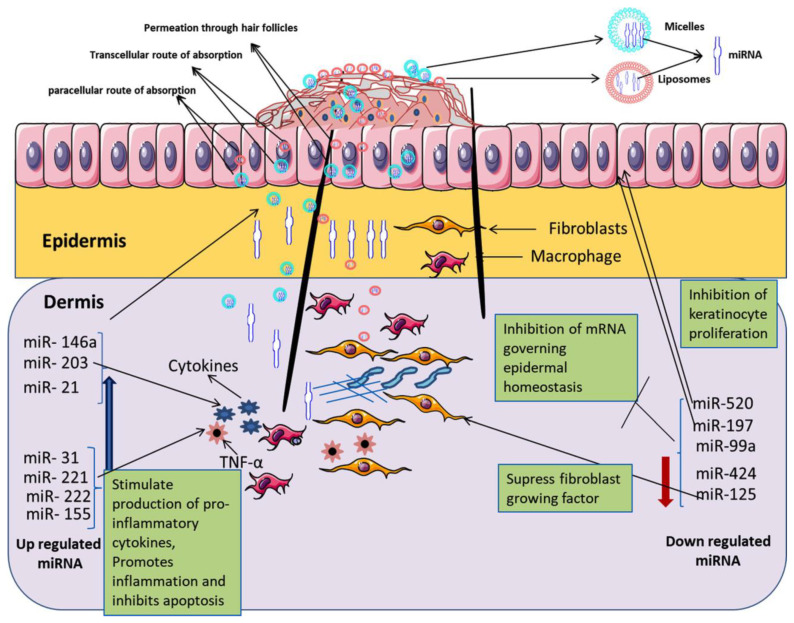
Schematic illustration shows liposomes and micelles mediated targeted topical pharmacotherapy employing miRNA to decrease the pathological conditions over the skin in psoriasis. Reproduced from Pradyuth Sai et al. [217], John Wiley and Sons, 2020.

It has been reported potential role of microRNA (miRNA) in the management of psoriasis opened the way for miRNA-based targeted pharmacotherapy in psoriasis. Pradyuth et al. suggested that the miRNA expression could be minimized as a potential biomarker to suppress the hyper-proliferation of keratinocytes and the production of inflammatory cytokine in the management of psoriasis [217]. The schematic illustration in Figure 8 highlights the delivery of miRNA through micelles/liposomes carrier system following the paracellular/transcellular route of absorption in skin and down-regulated epidermal homeostasis to achieve the goal of targeted pharmacotherapy through the inhibition of keratinocyte proliferation and inhibition of pro-inflammatory cytokine secretion.

### 6.4. Targeting through CD44 Receptors

**Figure 9:**
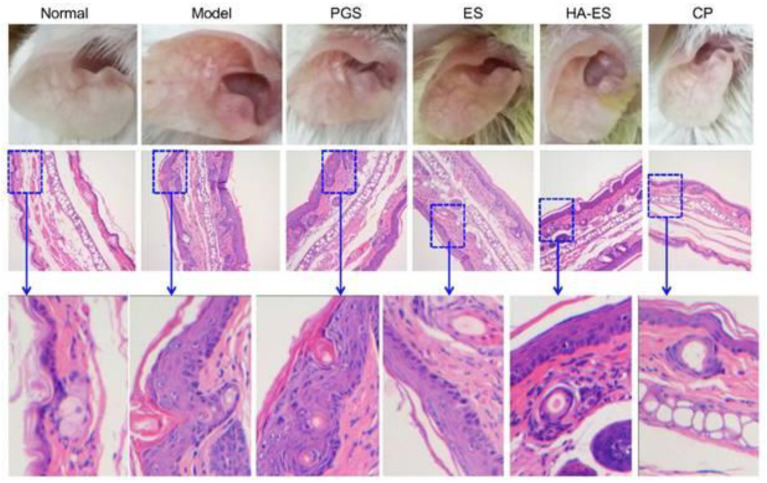
Illustration shows utilization of hyaluronic acid-modified ethosomes (HA-ES) for the delivery of curcumin by active targeting approach to the targeted site in psoriasis through CD44 receptor-mediated drug-delivery approach ultimately helpful to improve the therapeutic efficacy of curcumin compared to non-targeted ethosomes system (ES). Normal: No treatment; Model: Treated with imiquimod only to induce psoriasis; PGS: Treated with curcumin in propylene glycol solution; ES: Treated with curcumin-loaded ethosomes; HA-ES: Treated with curcumin-loaded hyaluronic-modified ethosomes; CP: Treated with clobetasol propionate marketed cream. Reproduced from Zhang Y et al. [220], Ivyspring International Publisher, 2019.

Lindqvist et al., in their investigation, described that the epidermis in psoriatic skin is overexpressed of CD44 receptor proteins and observed a significant reduction in hyaluronic acid distribution [218]. This receptor protein can be specifically targeted to enhance the efficacy of pharmacotherapy in psoriasis. Hyaluronic acid is a natural ligand for CD44 receptor proteins and Lv et al. reported the utilization of hyaluronic acid in targeted pharmacotherapy to improve the targeting of CD44 receptor proteins ultimately leading the drug deposition in disease sites and helping to minimize the untoward effects [219]. In another investigation, Yongtai Zhang et al. formulated curcumin-loaded hyaluronic acid-modified ethosomes to deliver the curcumin to the targeted site in psoriasis through CD44 receptor-mediated drug delivery [220]. The developed formulation system (HA-ES) was evaluated to observe the anti-psoriatic efficacy in an imiquimod-induced mouse model and compared the therapeutic efficacy of the non-targeted ethosomes system [220]. They reported that the HA-ES system effectively binds with CD44 receptor protein (abundant in inflamed psoriatic skin) for targeted delivery, thereby enhancing the accumulation of curcumin in inflamed psoriatic skin for better therapeutic efficacy compared to non-targeted ethosomes system (illustrated in Figure 9) [220]. 

## 7. Conclusive Remarks and Future Directions

The choice of therapy for the better management of psoriasis depends on several factors, such as disease severity, its consequence on a patient’s life, and the patient’s acuity for their disease. In terms of severity, there are three commonly employed therapeutic approaches in clinical practice, i.e., local therapy, phototherapy, and systemic therapy for the treatment of psoriasis. However, none of the currently employed therapeutic strategies are found to be potentially effective, safe, and able to completely cure psoriasis. Nowadays, antipsoriatic medicine prescribed in clinical practice is effective only to prevent the progression of the disease. The dearth of potentially effective and safer therapy for psoriasis has urged the need to design and develop nanomedicine-based therapy to make the treatment more promising and acceptable at a clinical level.

**Figure 10:**
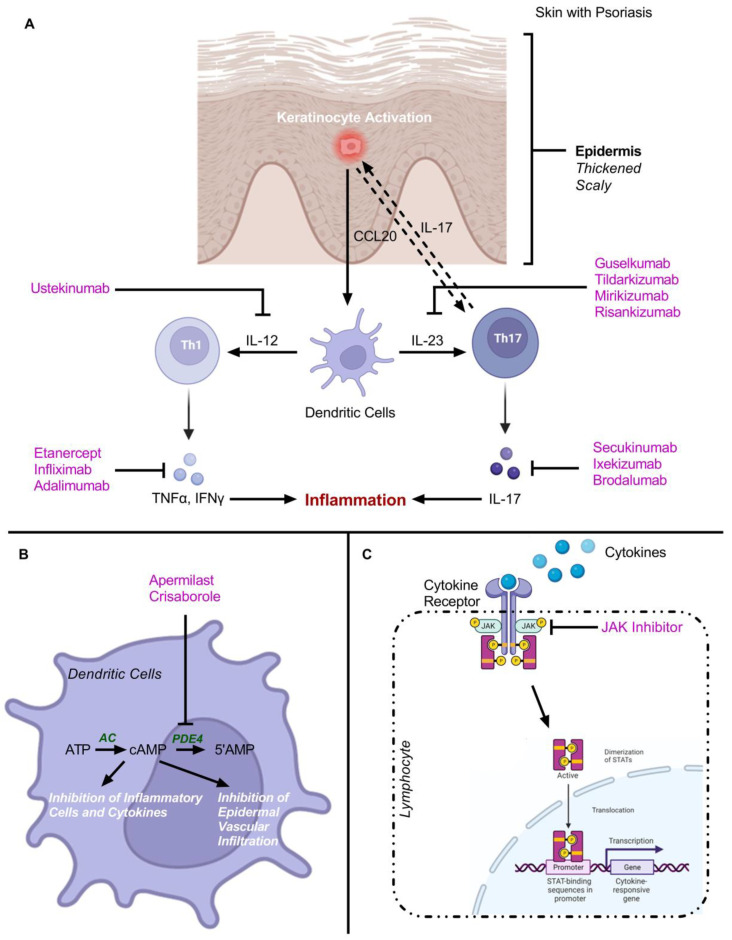
Schematic illustration highlights utilization of different biologics and small molecule therapeutics for targeting biomolecules to suppress inflammatory mediators and activated immune response for better management of psoriasis. (**A**) Different Biologics for cytokines inhibition. (**B**) Small molecule therapeutics as phosphodiesterase-4 (PDE-4) inhibitor. (**C**) Small molecule therapeutics as Janus kinases (JAK) inhibitors. Modified from Rapalli VK et al. [205], Elsevier, 2018. “Image created with BioRender.com”.

Nanotechnology-mediated drug delivery system (NDDS) plays a crucial role in the precise delivery of a variety of therapeutics including anti-psoriatic drugs. Nanomedicine is a relatively new era of nanobiotechnology; irrespective of that, the possibilities of this NDDS for pharmacotherapy seem endless. It has improved the therapeutic potential of drugs by reducing drug toxicity and/or enhancing the benefit/risk ratio by employing drug carriers-mediated delivery of loaded therapeutics including anti-psoriatic drugs. These novel intelligent systems can exploit the potency of therapeutics for psoriasis in great ways since they have the potential to promptly detect and respond to disease directly, without any impairment to a healthy cell or tissue and thereby improving the quality of a patient’s life. This novel approach is proficient in assuring the site-specific delivery of therapeutics across the skin to cure psoriatic lesions. Improved skin deposition with a targeting efficiency particularly in the epidermal/dermal layer is helpful in the success of nanomedicine. These smart delivery systems allow the administration of low doses and less dosing frequency for the same therapeutics compared to conventional drug delivery systems. In addition, the utilization of nanoscale topical pharmacotherapy for the delivery of biologics (illustrated in Figure 10) in combination with other small molecule therapeutics for targeting biomolecules to suppress the inflammatory mediators and activated immune response for better management of psoriasis is another promising strategy to achieve the aim for delivery of anti-psoriatic therapeutics for targeted pharmacotherapy. 

However, the strategy for its assessment and control of the interaction of nanomaterial in in vivo systems are some of the obstacles in translating these intelligent drug delivery systems to clinical practice. Targeting specific psoriatic lesions while circumventing the major organs (such as the spleen and liver) are major challenges that need to be addressed for its clinical translation. Clinical trials in this area should be bourgeoned to delineate the place of nanomedicine in the management of psoriasis. Extensive research and advancement in characterizing tools to determine the clinical safety and efficacy of nanomedicine-based therapy including nanoscale targeted pharmacotherapy for the management of psoriasis may become a reality in near future.

## Figures and Tables

**Table 1:** Available pharmacotherapy for the management of psoriasis.

Drug	Mechanism	Biopharmaceutical Attributes	Side-Effects	Ref.
Tacrolimus	-Inhibitory action on calcineurin phosphatase; -Suppress the expression of TNF-α e in monocytes through the MAPK-ERK pathway.	-Poor aqueous solubility; -Low permeation; -Bioavailability range from 11.2 to 19.1.	-Headache; -Flu-like symptoms; -Itching sensation.	[2,31,32]
Pimecrolimus	Macrophilin-12 (FKBP-12) and inhibits calcineurin; -Inhibits T-cell activation by inhibiting the synthesis and release of the cytokine from T-cells.	-High lipophilicity; -Insoluble in water; -Low permeation; -Low systemic absorption; -74–87% protein binding; -Undergoes hepatic metabolism.	-Burning, itching, redness, skin rash, swelling, or soreness at the application site.	[2,33,34]
Dithranol	Up-regulation of interleukin-10 receptor expression on keratinocytes	-0% bioavailability; -Absorbed and oxidized within the skin.	-Irritation of perilesional skin and staining of the skin and household items.	[2,35,36]
Vitamin D3 analogue	-Inhibits epidermal proliferation and promotes epidermal differentiation.	-5–6% bioavailability.	-Hypercalcemia; -Anorexia; -Pruritus.	[37]
Salicylic acid	-Intercellular bonding, swelling, and desquamation of corneocytes; -Hydration and softening of stratum corneum.	-Readily absorbed from the skin; -50–85% of protein binding.	-Skin irritation.	[2,38,39,40]
Urea	Antipruritic, epidermis-thinning, hydrating, hygroscopic, keratolytic, proteolytic, and penetration-enhancing effects.		-Skin irritation.	[38,41]
Corticosteroids Class I-*Clobestol propionate, Betamethasone dipropionate, Halobetasol propionate, Diflorasonediacetate * Class II-*Mometasonefuroate, fluocinoloneAcetonide* Class III-*Triamcinolone acetonide, Betamethasone valerate*	-Anti-inflammatory, immunosuppressant. -Broad range of anti-inflammatory, immunosuppressant, and vasoconstriction. -Inhibitory action of phospholipase A2 inhibitory protein (lipocortins).	-Metabolized in the liver and excreted via the kidney. -Dosage topical as 0.5–0.1% lotion/cream/ointment, apply 3–4 times daily. -Very low protein binding undergoes rapid metabolism	-Peroral dermatitis, Skin atrophy, hypertrichosis. -Skin thinning, skin atrophy, irritation, dryness, itching, folliculitis, hypertrichosis. -Folliculitis, dryness, irritation, hypertrichosis, acneiform eruptions.	[42,43,44] [45,46,47,48] [49,50]

**Table 2:** Available combination therapy for the management of psoriasis.

Combination Therapy	Drugs	Types of Psoriasis	Major Outcomes	Ref.
Calcipotriol and Corticosteroids	Calcipotriol/Betamethasone dipropionate	Stable psoriasis vulgaris	-Effective than tacalcitol in reduction in PASI.	[56]
	Calcipotriol/Betamethasone dipropionate	Psoriasis vulgaris	-The mean percentage reduction in PASI was more than 73 %.	[57]
	Calcipotriol/Betamethasone dipropionate	Severe plaque psoriasis	-75% of patients achieved a rating of bright or almost clear.	[34]
	Calcipotriol/Betamethasone valerate	Psoriasis vulgaris	-45% of patients show complete healing, and 32% show significant improvement.	[58]
	Calcipotriol/Clobetasol propionate	Severe plaque psoriasis	-Significant decrease in total symptom score.	[59]
	Calcipotriol/Clobetasol propionate	Stable plaque psoriasis	-50% reduction in the eruption score after two weeks. -Less adverse effects.	[60]
	Calcipotriol/diflucortolonevalerate	Chronic plaque psoriasis	-More rapid clinical response.	[61]
Calcipotriol and Retinoids	Calcipotriol/Acitretin	Chronic plaque psoriasis	-Enhanced clinical outcome of systemic acitretin. -Complete clearance.	[62]
	Calcipotriol/Acitretin	Plaque psoriasis	-Clearance or marked improvement was achieved by 67%. -Well tolerated and safe.	[63]
Calcipotriol and Cyclosporine	Calcipotriol/Cyclosporine	-Severe plaque psoriasis	-90% improvement in PASI. -More effective than placebo/cyclosporine.	[64]
Calcipotriol and Methotrexate	Calcipotriol/Methotrexate (MTX)	-Psoriasis vulgaris	-Safe and well tolerated. -Lower cumulative dosages of MTX.	[65]
Calcipotriene and Nicotinamide	Calcipotriene/Nicotinamide	-Mild to moderate psoriasis	-Clear to an almost clear outcome.	[66]
Corticosteroids and Tazarotene	Mometasonefuroate/Tazarotene	-Plaque psoriasis	-Better efficacy (in reducing plaque elevation) on trunk lesions.	[67]
	-Tazarotene/Fluocinonide 0.05% ointment, mometasone furoate, diflorasone diacetate 0.05% ointment). -Tazarotene/Betamethasone dipropionate, fluticasone propionate, diflorasonediacetate.	-Stable plaque psoriasis	-Within 8 weeks utmost improvements were recorded. -Tazarotene plus mometasone furoate was best tolerated therapeutic regimen.	[68]
Corticosteroids and Salicylic acid	Mometasonefuroate/Salicylic acid	-Psoriasis vulgaris	-Reduction in PASI score was 44%.	[69]
Corticosteroids and Retinoids	Triamcinolone acetonide/Etretinate	-Chronic plaque psoriasis	-Excellent improvement -Fewer side-effects.	[70]
Corticosteroids and biologic	Betamethasone with calcipotriol/Adalimumab	-Moderate to severe psoriasis	-Reduction in PASI score was 75%. -Well tolerated.	[71]
Phototherapy and Chemotherapy	UVB/Acitretin	-Psoriasis vulgaris	-Faster response. -Psoriatic disease cleared significantly.	[72]
	PUVA/Etretinate	-Severe psoriasis	-Significantly more effective than the other treatments. -Reduction in total UVA doses to about one-third.	[73]
	PUVA/Etretinate	-Guttate psoriasis	-Most effective outcome with 100% clearance rate.	[74]
	UVB/Methotrexate	-Chronic plaque	-More rapid clinical improvement compared.	[75]
	UVA1-UVB/Calcipotriol	-Plaque psoriasis	-Response to UVA1 and narrow-band UVB with Calcipotriol was better than Calcipotriol monotherapy.	[76]
	PUVA/Calcipotriol	-Psoriasis vulgaris	-Reduced cumulative dose and improved response to psoriasis vulgaris. -More than a 90% reduction in PASI score was recorded.	[77]
	NB-UV/Alefacept	Chronic plaque psoriasis	-Decrease (75%) in PASI score.	[78]
	UVB/Calcitriol	Plaque psoriasis	-The greater efficacy in combined treatment. -34% reduction in exposure to total UVB.	[79]
	Broadband UVB/Calcipotriol	Chronic psoriasis	-For the active group mean cumulative UVB dose was considerably lower. -Better reduction in PASI.	[80]
	Broadband UVB/Calcipotriol	Chronic psoriasis	-Safe and efficacious. -Resultant in limited exposure to UVB. -Time saver and limited cumulative irradiance.	[81]

**Table 3:** Lipid-based vesicular system (Liposome, ethosome, and noisome) utilized to improve the biopharmaceutical attributes/therapeutic efficacy of encapsulated drugs/actives in psoriasis.

Carrier	Drug	In Vitro/In Vivo Model	Outcomes	Ref.
Liposome	Fusidic acid	-Mouse tail model.	-Enhanced cellular uptake. -Improved efficacy.	[84]
Liposome	Psoralen	-Imiquimod-induced psoriatic plaque model.	-Reduced psoriatic symptoms. -Reduction in TNF-α, IL-17 and IL-22.	[86]
Ethosomes	Psoralen	-Human embryonic skin fibroblasts.	-Improved dermal and transdermal delivery. -Enhanced bioavailability.	[87]
Liposome	RNA interference	-Intact human skin.	-Down-regulation of human beta-defensin 2.	[88]
Liposome	Plasmid DNA	-K14-VEGF transgenic mouse.	-Enhanced anti-psoriatic effect. -Suppressed hyperplastic and inflamed vessels.	[89]
Liposome	Methotrexate	-Porcine skin.	-Enhanced skin permeability.	[90]
Liposome	Methotrexate	-Albino rat.	-Enhanced permeability.	[91]
Ethosomes	Methotrexate	-Human cadaver skin.	-Enhanced permeation in deeper skin layers. -Storage stability and formulation retained their efficiency.	[92]
Liposomal lotion	Tacrolimus	-C57BL/6 mice.	-Higher skin concentrations than systemic. -Better efficacy in the prevention of delayed hypersensitivity reactions.	[93]
Liposome	Dithranol	-Mouse abdominal skin.	-Enhanced skin permeation.	[94]
Liposome	Dithranol	-Human volunteer.	-Total clearance of lesions. -No reports of lesion or perilesional irritation.	[95]
Liposome	Calcipotriol	-Rat abdominal skin.	-Augmented deposition of calcipotriol in stratum corneum layer	[96]
Liposphere	Thymoquinone	-BALB/c mice. -RAW 264.7 cells.	-Deeper skin penetration. -Improved disease symptoms, supported by reduced ultrastructural changes, IL-17 and TNF-α in skin lesions. -Diminution of nitric oxide, IL-1β, TNF-α, IL-2, and IL-6.	[97]
Lipogel	Cyclosporine	Human volunteer with chronic plaque psoriasis	-After eight weeks, a significant reduction (approximately 83%) in dermatological sum score (DSS) was observed. -Complete clearance/recovery at the end of the study (i.e., DSS = 0).	[102]
Liposome	Dithranol	Human volunteer with bilaterally symmetrical stable plaque psoriasis	-Significantly reduced total severity score was observed.	[103]
Niosomes	Diacerein	-Rat skin.	-Enhanced skin deposition.	[107]
Niosomes	Tazarotene	-Rat skin.	-Enhanced local bioavailability. -Higher skin retention within the skin layers.	[108]
Niosomes	Capsaicin	-Rat skin.	-Significantly higher accumulation of the drug.	[109]
Niosomes	Combination of methotrexate and cyclosporine	-Mouse	-Reduced the systemic side effects -Good healing of psoriasis	[110]
Nanosomes (Secosomes)	siRNA	-Bioengineered skin-humanized mouse.	-Significant improvement in the psoriatic phenotype. -Improvement in filaggrin expression, transglutaminase activity, and appearance of stratum corneum.	[111]
Liposome	Tamoxifen	-Mice.	-Significantly enhanced efficacy of Tamoxifen.	[112]
Niosomes	Glycyrrhizinate	-Human dermal fibroblasts. -Murine model.	-Decreased edema and nociceptive responses.	[113]
Niosomes	Methotrexate	-Human volunteer.	-Significant reduction in total score (from 6.2378+/−1.4857 to 2.0023+/−0.1371).	[116]
Liposome	Methotrexate	Albino mice	-Enhanced antipsoriatic activity. -No systemic toxicity.	[118]
Liposphere	Tacrolimus and Curcumin	-BALB/c mice.	-Improved skin deposition. -Diminution of TNF-α, IL-17 and IL-22 levels.	[119]
Niosomes	Methotrexate	-Albino rat.	-Prominent accumulation of the drug in the skin. -Safely topical applied.	[120]

**Table 4:** Lipid-based nanoparticulate system (SLN, NLC) utilized to improve the biopharmaceutical attributes/therapeutic efficacy of encapsulated drugs/actives in psoriasis.

Carrier	Drug	In Vitro/In Vivo Model	Outcomes	Ref.
NLC	Fluocinolone acetonide	Rat skin	-Prominent uptake of drug in dermal/epidermal layers. -Preferred drug retention in the epidermis and reduced adverse effects connected with systemic exposure.	[36]
SLN	Tretinoin	-Abdominal rat skin.	-Significant improvement in photostability. -Less irritation to the skin -Improved topical delivery of the drug.	[121]
SLN	Betamethasone dipropionate Calcipotriol	-Mouse. -HaCaT cells.	-Decreased epidermal thickness. -Increased melanocyte count. -Negligible skin irritation. -Better skin tolerability. -Delayed abrupt growth of keratinocytes.	[126]
SLN	Halobetasol propionate	-Human cadaver skins.	-Improved skin deposition. -Improved skin uptake and nonirritant.	[127]
SLN	Mometasone furoate	-Rat skin.	-Enhanced skin deposition.	[128]
SLN	Triamcinolone acetonide	-Abdominal rat skin.	-Improved skin deposition.	[129]
Lipid NPs (SLN, NLC)	Capsaicin	-Rat skin.	-Improved drug accumulation in skin layers.	[130]
SLN	Cyclosporine A	Murine model	-Improved skin penetration. -Decreased level of T helper (Th) 2, IL-4, and -5.	[132]
NLC	Acitretin	-Human cadaver skin.	-Significantly higher skin deposition of Acitretin. -Significant improved therapeutic response.	[142]
NLC	Methotrexate	Rat skin	-Higher skin penetration.	[143]
NLC	Methotrexate	Mice	-Prominent diminution of PASI score with recovery skin in mice.	[144]
NLC	Calcipotriol with Methotrexate	Mice	-Enhanced permeation in hyperproliferative skin.	[145]
NLC	Tacrolimus	-Guinea pig.	-Less irritating. -Improved skin deposition.	[147]
SLN	Clobetasol propionate	-Human volunteer.	-Significant reduction in inflammation and itching symptoms as compared to marketed preparation.	[155]

**Table 5:** Polymeric nanoparticulate system utilized to improve the biopharmaceutical attributes/therapeutic efficacy of encapsulated drugs/actives in psoriasis.

Carrier	Drug	In Vitro/In Vivo Model	Outcomes	Ref.
Polymeric micelle	Tacrolimus	-Mice.	-Improved drug accumulation in skin layers.	[149]
Polymeric NPs	Dead Sea Minerals(DSW)	-In vitro.	-High drug loading.	[160]
Polymeric NPs	Curcumin	-Imiquimod-induced psoriatic mice.	-Higher therapeutic effect. -More powerful skin-permeating capability. -Reduction in expression of IL-6, TNF-α, NF-κB.	[161]
Polymeric NPs	Curcumin	-Human volunteer.	-Significantly improved skin permeability. -Significantly higher reduction in PASI.	[162]
Polymeric NPs	Dithranol	-Ex vivo rat skin.	-Improved topical bioavailability.	[165]
Hybrid polymer-lipid NPs (PLNs)	siRNA	-Imiquimod-induced psoriatic mouse model.	-Developed a system helpful to combine silencing gene therapy and photochemical internalization for topical treatment in psoriasis.	[166]
pH-sensitive polymeric NPs	Methotrexate	-Imiquimod-induced psoriatic mice model.	-Developed system exhibited negligible signs of hyperkeratosis and parakeratosis.	[167]
Lecithin-chitosan hybrid NPs	Tacrolimus	-Imiquimod-induced psoriatic mouse mode.l	-Significant improvement in drug deposition in skin and in vivo anti-psoriatic activity of developed system compared to the marketed product.	[168]
Polycaprolactone NPs	Hydrocortisone	-HaCaT cell lines.	-Developed system exhibited negligible toxicity to the keratinocyte cells compared to pure drug.	[169]
Chitosan/hyaluronic acid NPs	Alantolactone	-Imiquimod-induced psoriatic mice model.	-Developed system minimized the STAT3 hyperactivation within keratinocytes and improved psoriatic signs/symptoms.	[170]
Albumin NPs	Methotrexate	-Imiquimod-induced C57BL/6 mice.	-Developed system significantly decreased the erythema and minimized skin thickness. -Significantly ability for targeted delivery of methotrexate to a lymph node.	[171]

## Data Availability

Not applicable.

## Abbreviations

Act	Acitrein
AE	Aloe-emodin
AgNPs	Silver nanoparticles
CLSM	Confocal laser scanning microscopy
Cs	Cyclosporine
CT	Calcipotriol
DCs	Dendritic cells
DDS	Drug delivery systems
DSW	Dead Sea Water
GNPs	Gold nanoparticles
hBD-2	Human β defensin-2.
IL	Interleukin
IT	Isotretinoin
KGF	keratinocytes growth factor.
MNPs	Metallic nanoparticles.
MTX	Methotrexate.
NEG	Nanoemulgel
NLCs	Nanostructured Lipid Carriers.
NPs	Nanoparticles
NSAIDs	Nonsteroidal anti-inflammatory drugs
PASI	Psoriasis area and the severity index
PDGF	Platelet-derived growth factor
PM	Polymeric micelles
PNPs	Polymeric nanoparticles
TAM	Tamoxifen
Th cell	T helper cell
TNF	Tumor necrosis factor
ToPAS	Toronto psoriatic arthritic screening
TRE	Tretinoin
UCL	Ultra deformable cationic liposome
ZnONPs	Zinc oxide nanoparticles
